# Book Review

**DOI:** 10.1177/03010066211041231

**Published:** 2021-08-30

**Authors:** Greig Dickson

**Affiliations:** School of Psychological Science, University of Bristol, Bristol, UK

Written as part of the Cambridge Elements Perception series, Brett R. Fajen's Visual Control of Locomotion provides a comprehensive overview of decades of research examining the role of visual perception in the control of self-motion, specifically human locomotion. The principal drive behind his contribution is to further promote the concept that perception and action are inextricably intertwined, and that the predominant role of visual perception is to guide movement in dynamic and structurally complex environments.

Fajen creatively employs the use of ecologically relevant and relatable imagery throughout the book to synthesise and elucidate often complex concepts, accentuating the key theoretical gains made within the field since Gibson's seminal theory of affordances (Gibson, 1977). For instance, Fajen opens the introduction with his first of many highly descriptive, real-world examples of the symbiotic nature of visual perception and action. Here, the reader is narrated through a cross-country run in which a creek is leapt, a large tree-limb is hurdled, and a densely wooded forest is traversed before a road is successfully navigated across, whilst avoiding an oncoming car. Through this description, Fajen clearly exhibits the interdependent relationship between visual perception and action, highlighting the key areas that he proceeds to discuss in the subsequent sections. This clarity of thought is reflected throughout the book, where Fajen successfully delivers a work that is as accessible to graduate students as it is to experts in the field, whilst managing to avoid diluting or over-simplifying the content.

Section two of the book provides a detailed account of the prominent role of optic flow in how we determine self-motion during locomotion. This is centred around the perception of speed, heading and path from the expanding flow field that is created as we move through any structured environment. What is of particular interest here, is when the discussion delves into perceived heading direction during rotation and curvilinear self-motion. This is followed by an insightful treatment of retinal flow in shaping our perception of a future path and interpreting object-motion during self-motion. Concluding with a subsection on time-to-contact and on the neuro-mechanical underpinnings of optic flow and self-motion, Fajen provides a brief yet helpful synopsis of research examining the potential neural mechanisms of self-motion perception, thus far.

The third section examines the embodiment of spatial mapping, contextualised by how we perceive the spatial layout of a scene and determine the affordances available to us, given the specifics of the environment and the functionality of our unique bodies. Here, Fajen provides an overview of Gibson's theory of affordances (Gibson, 1966, 1977, 1979), where our perception of the physical environment is scaled by our body and physical capabilities. Affordance grows in complexity throughout development as we modify our relationship with the external environment in relation to our body. With every new experience, we continually update our understanding of the affordances that our bodies facilitate and our perceptions of the world around us. This adaptive strategy is cleverly demonstrated by Fajen's reference to the character of Jake in the movie, Avatar (Cameron, 2009). Jake embodies an avatar to move more effectively through the new world that he is about to inhabit. This physical adaptation is initially strewn with erroneous movements, due to the increased size of the avatar in comparison to his human body. However, he quickly adjusts to, and masters the movement of, the avatar. By using this analogy, Fajen reveals his skill in delivering accessible examples to illustrate his point. In this instance, he asserts the adaptability of humans to the affordances of their physical body, providing an egocentric, sensory connection to the surrounding environment. This section, detailing the concept of affordances, neatly concludes the first half of the book that focuses on the role of visual perception in guiding locomotion.

Section four moves on to discuss the online, goal-directed approach of how vision is used to control locomotion. Here, information-based control models of action are examined, which are later compared with internal model-based approaches in section five. The information-based models presented propose that only specific information is required to inform action and alter movement, including perceptual information available in that moment (within the visual scene), and task-specific information. Additionally, the information-based models described infer that behaviour is emergent in real-time as a response to this information, and so is not pre-planned. Fajen goes on to investigate information-based models that adhere to the current-future control strategy – a predictive approach to action based on what would happen if current conditions continued over time. Again, he uses real-world examples, such as catching a baseball (intercepting a moving target) and bringing a moving vehicle to a stop at an intended location, to render his point. Developing the discussion from previous sections, Fajen also explores affordance-based models of control – a strategy which supposes that an agent's movements are guided by the potentials for action given the specific circumstances at that moment. Similar ground-based, aerial-based, and collision-avoidance examples are used to examine these models. This deftly leads to a subsection of particular interest: Fajen's comparison of current-future control models and affordance-based control models of action. The discussion settles on the key limitation that current-future models treat visual guidance and action selection as different processes – two processes which are fully integrated within affordance-based models. This section is concluded with an evaluation of Warren's behavioural dynamics framework (Warren, 2006), extending Gibson's theory of affordances (Gibson, 1977), where agent and environment are regarded as two dynamic systems, synergised by information and action. Warren's framework is considered in relation to steering, obstacle avoidance, and route selection, before finally being applied to an examination of how we navigate crowds.

The final section introduces the debate around internal models, regarding the visual control of locomotion. Having discussed online, information-based models of control in the previous section, Fajen proceeds to examine models of control where visual information is not available, such as when a moving object is occluded. Fajen establishes two fundamental properties of an internal model: that the model emulates occurrences in the external world, and that it serves as a replacement for some external situation or condition. After conceding to the consensus that both information-based models and internal models are not considered to collectively function as an explanation for how an action is guided in different contexts (i.e. an information-based model when visual information is available, and an internal model when visual information is not), Fajen contemplates whether either model can entirely replace or account for the other. He then moves on to explore the evidence concerning the sufficiency of internal models to account for behaviour, both in the absence of information, and with respect to the role of such models when visual information is available. He is careful not to choose sides here, and simply presents the available evidence. This reflects the book's functionality as an educational tool that consolidates the key research within this subject area, whilst raising open questions of relevance and interest.

Fajen goes on to express his expectation that technological advances will facilitate the study of the role of visual perception in controlling locomotion in more ecologically valid settings; something that is currently very much taking place – particularly regarding motion-tracking and mobile eye-tracking. As Fajen concludes in the closing paragraph, these advances promise to further contribute to a growing field that has implications for the future design of semi-autonomous vehicles, and for aiding mobility and furthering the sensory experience of people with visual and motor impairments.

In writing this Cambridge Elements book, Fajen employs his rich and detailed knowledge, to depict the relationship between perception and action in the visual control of locomotion. Through the clear structure and writing, and the organisation and presentation of his arguments into sensible and easy to navigate sections, he has produced a piece of highly accessible work that is an excellent resource for graduate students and a valuable read for academics with an interest in the visual control of locomotion.
